# Hybrid repair of a very late, post-aortic coarctation surgery thoracic aneurysm: a case report

**DOI:** 10.1186/1752-1947-6-255

**Published:** 2012-08-30

**Authors:** Ioan Tilea, Laszlo Hadadi, Razvan C Serban, Brindusa Tilea

**Affiliations:** 1 Emergency Clinical County Hospital, Cardiac Catheterization Laboratory, 50 Gheorghe Marinescu St, Targu Mures, 540136, Romania; 2 University of Medicine and Pharmacy of Targu Mures, 38 Gheorghe Marinescu St, Targu Mures 540139, Romania; 3 Emergency Clinical County Hospital, Cardiology Clinic I, 50 Gheorghe Marinescu St, Targu Mures, 540136, Romania; 4 Emergency Clinical County Hospital, Department of Infectious Diseases, 50 Gheorghe Marinescu St, 540136, Targu Mures, Romania

**Keywords:** Aortic aneurysm, Aortic coarctation, Aortic stent-graft

## Abstract

**Introduction:**

Local aneurysms after surgical repair of coarctation of the aorta occur mainly in patients surgically treated by Dacron patch plasty during adulthood. The management of these patients is always problematic, with frequent complications and increased mortality rates. Percutaneous stent-graft implantation avoids the need for surgical reintervention.

**Case presentation:**

We report a case involving the hybrid treatment by stent-graft implantation and transposition of the left subclavian artery to the left common carotid artery of an aneurysmal dilatation of the thoracic aorta that occurred in a 64-year-old Caucasian man, operated on almost 40 years earlier with a Dacron patch plasty for aortic coarctation. Our patient presented to our facility for evaluation with back pain and shortness of breath after minimal physical effort. A physical examination revealed stony dullness to percussion of the left posterior thorax, with no other abnormalities. The results of chest radiography, followed by contrast-enhanced computed tomography and aortography, led to a diagnosis of giant aortic thoracic aneurysm. Successful treatment of the aneurysm was achieved by percutaneous stent-graft implantation combined with transposition of the left subclavian artery to the left common carotid artery. His post-procedural recovery was uneventful. Three months after the procedure, computed tomography showed complete thrombosis of the excluded aneurysm, without any clinical signs of left lower limb ischemia or new onset neurological abnormalities.

**Conclusions:**

Our patient’s case illustrates the clinical outcomes of surgical interventions for aortic coarctation. However, the very late appearance of a local aneurysm is rather unusual. Management of such cases is always difficult. The decision-making should be multidisciplinary. A hybrid approach was considered the best solution for our patient.

## Introduction

Coarctation of the aorta is a relatively frequent congenital cardiovascular disease, accounting for approximately 4% of all congenital cardiovascular abnormalities [1]. The usual approach is the surgical correction of the anomaly. Despite high initial success rates, more than 9% of patients develop late complications, such as systemic hypertension, premature coronary artery disease, aortic valve abnormalities, aneurysm formation, or recoarctation [2,3]. Post-surgical aneurysms are described after subclavian flap angioplasty in 17% of cases, patch angioplasty in 14% of cases, interposition graft repair in 6% of cases and, occasionally, after end-to-end anastomosis in patients with persistent systemic hypertension [4,5]. Post-surgical aneurysms are particularly frequent after Dacron patch plasty performed in adulthood. Late occurrence of aortic aneurysms after surgical correction of aortic coarctation carries a significant risk of rupture and is associated with high mortality rates. Management of such patients is always challenging. Conservative treatment has unpredictable short-term results. A single-center study reported a 100% rate of rupture within 15 years [6]. Surgical reinterventions require a complex approach, including circulatory arrest, frequent use of blood products and a prolonged hospital stay. Moreover, surgical revision is associated with significant morbidity by paraplegia, injury of the central nervous system, or bleeding and 14% in-hospital mortality rates [7-9]. The use of balloon-expandable endovascular stents has been previously reported as successful in both primary treatment of coarctation and recoarctation of the aorta [9,10]. This technique also makes it possible to avoid a major surgical procedure and its inherent risks [1,11,12].

Ince *et al*. reported no mortality and minimal morbidity in a series of six patients treated by percutaneous techniques and endovascular graft implantation for aneurysms occurring after previous repair of an aortic coarctation [1]. These results are significantly superior to those seen with open surgical treatment, which have 14% to 23.5% mortality rates [6,13].

## Case presentation

Almost two years ago, a 64-year-old Caucasian man was referred to our Cardiology Department for evaluation of back pain and shortness of breath on minimal physical activity. He had a long medical history, including surgical repair of aortic coarctation by Dacron patch plasty at the age of 26, with residual systemic hypertension. He also underwent bi-leaflet prosthetic valve (Sorin Biocarbon 27) implantation for severe degenerative stenosis of the bicuspid aortic valve at the age of 54. Twelve years previously he had been referred to hospital for ischemic stroke with left hemiparesis. On that occasion he was diagnosed as having a 5cm diameter asymptomatic aneurysmal dilatation of the thoracic aorta at the site of the first surgical procedure. Given the lack of symptoms at that time, the decision was made to follow him with regular reassessments.

Twelve years later, given the onset of symptoms, he was hospitalized in our department. At admission, physical examination revealed stable vital signs, blood pressure at 160/80mmHg, normal heart rate at 64 beats per minute, stony dullness to percussion of the left posterior thorax, and no evidence of left ventricular dysfunction at rest. An electrocardiogram showed sinus rhythm, first-degree atrioventricular block with a PQ interval of 280 milliseconds, left bundle branch block and rare ventricular premature beats. Conduction abnormalities had been already documented during previous evaluations.

**Figure 1:**
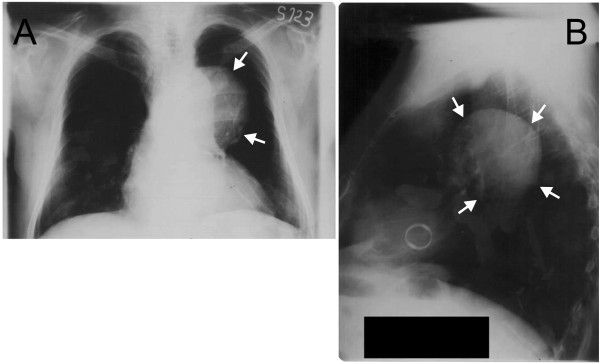
Chest radiography: antero-posterior (A) and left latero-lateral (B).

**Figure 2:**
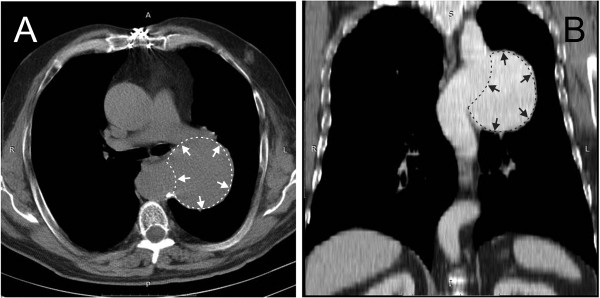
**Contrast-enhanced computed tomography (CT) scan: transversal section (A) frontal plane bi-dimensional reconstruction (B).** Image shows a 91×80×71mm aneurysm of the descending aorta.

Initial evaluation consisted of a plain chest radiograph, which showed a well-contoured opacity with a diameter of approximately 10cm, next to the left border of the aorta (Figure1A,B). The chest contrast-enhanced computed tomography (CT) scan confirmed the presence of a large dilatation of the thoracic aorta (Figure2A,B). Transthoracic echocardiography found good left ventricular systolic function, normofunctional aortic prosthesis, no abnormalities of the native valves, absence of pulmonary hypertension, absence of pericardial effusion, and a dilated (49mm) ascending aorta and aneurysmal descending thoracic aorta. Coronary angiogram and aortography performed in the same session showed no significant lesions of the epicardial vessels and location of the aortic aneurysm immediately below the left subclavian artery.

A multidisciplinary team, including a cardiologist, cardiovascular surgeon, angiographist and radiologist, decided on a hybrid resolution of the aneurysm.

**Figure 3:**
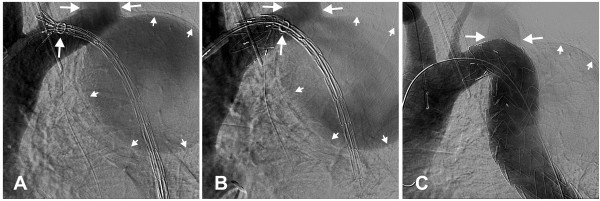
**Angiography with stent-graft: positioning (A), and implantation (B).** Immediate post-intervention angiography shows minimal leakage to the dilated left subclavian artery (**C**).

**Figure 4:**
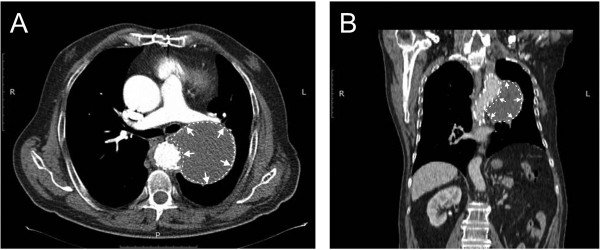
**Three-month follow-up chest computed tomography (CT) scan: complete thrombosis of the aneurysm, without leakage from the stented segment.** Transversal section (**A**), and frontal plane bi-dimensional reconstruction (**B**).

One year after the last evaluation successful exclusion of the aneurysm was carried out by percutaneous stent-graft implantation (Proximal Free Flow Straight Valiant Thoracic® TF3232C200X, Medtronic Inc., Minneapolis, MN, USA) using a right femoral approach (Figure3A,B). The stent-graft partially covered the ostium of the left subclavian artery and a paraprosthetic leakage was observed (Figure3C). The percutaneous intervention was continued with the surgical transposition of the left subclavian artery to the left common carotid artery within the same session. The post-procedural debranching had to be done because of the occlusion of the left subclavian artery during the procedure. Pre-procedural evaluation had not suggested this, but at the end of the interventional procedure the final angiogram revealed a partially covered ostium of the left subclavian artery by the stent-graft. The surgical possibilities for debranching in this situation were carotid-subclavian bypass or transposition of the left subclavian artery to the left common carotid artery. We chose the second procedure because of the presence of a paraprosthetic leakage originating from the left subclavian artery. The closure of the subclavian artery with sutures resolved both problems: the leakage and the left upper arm and vertebral artery vascularization. Both procedures were successful. Post-procedural evolution was uneventful, except for a fever of a few days duration of up to 38°C, which was related to post-implantation syndrome, with no evidence of infection and mild residual dysphonia. Three months after the procedure, our patient was clinically well without any clinical signs of left lower limb ischemia or new onset neurological abnormalities. Contrast-enhanced CT revealed complete thrombosis of the excluded aneurysm (Figure4A,B).

## Discussion

We present a case that illustrates the clinical outcomes of surgical intervention for aortic coarctation: residual systemic hypertension, aortic valve disease requiring surgical intervention, and dilatation of the ascending aorta, as well as aneurysm formation at the site of the surgical anastomosis, all occurring after Dacron patch plasty. However, such a very late presentation and slow evolution of the local aneurysm are rather unusual. The management of such cases is always difficult, requiring a multidisciplinary approach. Such a large aneurysm is prone to rupture when conservatively treated, but a third surgical intervention would have been of particularly high risk. In this case, a multidisciplinary team decided on a hybrid approach, which confirmed once again the safety and feasibility of this procedure in high-risk patients. Thus, non-surgical reconstruction of late, post-aortic coarctation surgery thoracic aneurysms appears a safe and feasible technique. Interventional stent-graft placement allows avoiding repeated surgical interventions.

There are no clear guidelines, however, regarding the best treatment of aortic aneurysm occurring after surgical correction of aortic coarctation. Recent advances in surgical management offer better results from surgical repair in the hand of experts. However, such procedures cannot avoid relatively long circulatory arrest, frequent pulmonary complications, a need for blood products in more than 50% of the cases and a prolonged hospital stay [7,8]. Conversely, percutaneous repair in such challenging cases requires less procedural time and no cardiac arrest. Thus, the interventional placement of stent-grafts offers the possibility of avoiding the risks associated with repeated surgery [1]. This case underscores the need for a multidisciplinary approach in the management of complex aortic diseases.

## Conclusions

This case illustrates clinical outcomes of surgical interventions for aortic coarctation in a patient with the very late appearance of a local aneurysm. Clinical decision-making and treatment in this unusual situation should be multidisciplinary and a hybrid approach considered with percutaneous stent-graft implantation.

## Consent

Written informed consent was obtained from the patient for publication of this case report and any accompanying images. A copy of the written consent is available for review by the Editor-in-Chief of this journal.

## Competing interests

The authors declare that they have no competing interests.

## Authors’ contributions

IT performed the interventional procedure and drafted the manuscript. BT, RCS, and LH assured the follow-up of our patient, helped in drafting the manuscript and made critical revisions to the manuscript. All authors read and approved the final manuscript.
